# Brown Planthopper (*N. lugens* Stal) Feeding Behaviour on Rice Germplasm as an Indicator of Resistance

**DOI:** 10.1371/journal.pone.0022137

**Published:** 2011-07-14

**Authors:** Mohamad Bahagia AB Ghaffar, Jeremy Pritchard, Brian Ford-Lloyd

**Affiliations:** School of Biosciences, University of Birmingham, Birmingham, United Kingdom; French National Centre for Scientific Research, Université Paris-Sud, France

## Abstract

**Background:**

The brown planthopper (BPH) *Nilaparvata lugens* (Stal) is a serious pest of rice in Asia. Development of novel control strategies can be facilitated by comparison of BPH feeding behaviour on varieties exhibiting natural genetic variation, and then elucidation of the underlying mechanisms of resistance.

**Methodology/Principal Findings:**

BPH feeding behaviour was compared on 12 rice varieties over a 12 h period using the electrical penetration graph (EPG) and honeydew clocks. Seven feeding behaviours (waveforms) were identified and could be classified into two phases. The first phase involved patterns of sieve element location including non penetration (NP), pathway (N1+N2+N3), xylem (N5) [21] and two new feeding waveforms, derailed stylet mechanics (N6) and cell penetration (N7). The second feeding phase consisted of salivation into the sieve element (N4-a) and sieve element sap ingestion (N4-b). Production of honeydew drops correlated with N4-b waveform patterns providing independent confirmation of this feeding behaviour.

**Conclusions/Significance:**

Overall variation in feeding behaviour was highly correlated with previously published field resistance or susceptibility of the different rice varieties: BPH produced lower numbers of honeydew drops and had a shorter period of phloem feeding on resistant rice varieties, but there was no significant difference in the time to the first salivation (N4-b). These qualitative differences in behaviour suggest that resistance is caused by differences in sustained phloem ingestion, not by phloem location. Cluster analysis of the feeding and honeydew data split the 12 rice varieties into three groups: susceptible, moderately resistant and highly resistant. The screening methods that we have described uncover novel aspects of the resistance mechanism (or mechanisms) of rice to BPH and will in combination with molecular approaches allow identification and development of new control strategies.

## Introduction

Rice, one of the world's most important food crops is attacked by insect pests totalling around 800 species, in both field and storage [1]. One of the most economically important insects is the brown planthopper (BPH) which can cause huge destruction of plants. China and Vietnam, two of the largest rice producing countries, suffered large production losses due to BPH attack in 2005 and 2006 [2]. BPH damaged plants directly by removal of plant sap but also indirectly by transmission of rice viruses such as ragged stunt virus and grassy stunt virus [3], [4].

Extensive chemical control of BPH on rice can cause serious problems including toxicity to natural enemies of BPH such as *Anagrus nilaparvatae* [5], increased total production cost, and possible long term agro-ecosystem and human health damage [6], [7]. Breeding programmes to develop rice varieties resistant to insect pests should therefore complement or replace conventional control strategies. More than 19 major BPH resistance loci (*bph1* to *bph19*) have already been identified in rice cultivars and wild species [8], [9], [10], [11], [12], [13], [14], [15], [16], [17] located on at least 5 different chromosomes.

Some of these resistance loci have already been successfully used as parents for breeding programs, and include rice varieties Mudgo (*bph1*), ASD7 (*bph2*), Rathu Heenathi *(bph3*) and Babawee *(bph4*) [18], [19].

Although many resistance loci have already been discovered, not all can be used to protect the rice plant from BPH attack [8]. At the heart of the problem is the ability of sap-feeding insects to overcome the many adaptations that plants have evolved as protection. The complex interaction between sap-feeding pests and their host plants has only recently begun to be understood, and it is clear that the pathway from host location to sustained ingestion of phloem sap can be interrupted at several points, potentially allowing many different types of resistance. Detailed comparison of the similarities and differences in the feeding behaviour of BPH on different rice genotypes varying in resistance will allow underlying mechanisms to be identified providing new targets for control.

The mouthparts of BPH, like other phloem feeding insects, consist of a stylet bundle which forms the piercing and sucking organ [20], [21]. BPH feeds on the plant by inserting the stylet bundle with an accompanying salivary sheath into the plant [22] locating the phloem tissue and then regulating the ingestion of the pressurised plant sap [20], [21]. Hattori [23] suggested that the BPH feeding process could be divided into two main phases. The first phase involves the movement of stylet tip across the plant tissue, while the second phase involves the feeding process [23] where the stylets enter vascular bundles and ingest the phloem sap. BPH feeding processes are complex but the use of the electrical penetration graph (EPG) technique [24] provides an opportunity for detailed cataloguing of stylet activities during feeding [25].

Several studies have previously investigated BPH feeding behaviour using this technique [3], [21], [23], [26], [27], [28]. These studies have correlated EPG waveforms with particular BPH stylet activities, and each study has made its own characterization. The method was first used by McLeans and Kingsley [29] which was AC (alternative current)-based, and it was subsequently improved by Tjallingii [24] using DC (direct current). Recent studies provide increasing levels of signal detail (e.g. Kimmins [26], Seo *et al* [21]). The present study exploits the EPG capability by using the DC-EPG technique to compare BPH feeding patterns and so host plant resistance across a range of rice genotypes. In common with other recent studies we have characterised our wave forms following the descriptions provided by Seo e*t al* [21].

## Results

### Rate of Honeydew Production

BPH feeding on IR694 demonstrated both the highest total number of honey dew droplets and highest average number per h with 104.3 droplets and 8.9 droplets per h respectively (table 1). BPH feeding on TN1 showed the shortest time to first honeydew production, producing droplets 4 h after introduction to the plant. BPH feeding on Azucaena, IR694 and Nipponbare were similar to TN1. In contrast, Rathu Heenathi did not produce a single honeydew drop over the whole 12 h of the experiment, while IR64, Babawee and F1also produced only a very low amount of honeydew. BPH took more than 8 h to produce honeydew on IR64, Babawee, F1 and MR232.

**Table 1:** Honeydew production over 12 h by *N. lugens* on 12 rice varieties using the honeydew clock method.

	N	Total honeydew droplets in 12 hours ± SE	Average honeydew droplets per hour± SE	Fastest time honeydew produce (hour)
**Azuceana**	8	79.2 abc (±15)	7.8 ab (±1.3)	4.3 g (±0.7)
**Nipponbare**	12	57.4 cd (±10.6)	5.0 c (±0.9)	5.7efg (±0.9)
**TN1**	13	90.7 ab (±11.1)	7.8 ab (±0.9)	4.0 g (±0.6)
**IR694**	11	104.3 a (±15.6)	8.9 a (±1.3)	4.5fg (±0.9)
**Fujisaka**	10	66.8 bcd (±20.8)	5.6 bc (±1.7)	6.8def (±1.0)
**IR758**	11	43.1 de (±15.9)	3.7 cd (±1.3)	7.9cde (±1.1)
**MR232**	10	16.1 ef (±7.9)	1.3 de (±0.7)	9.7abc (±1.0)
**MR219**	14	40.5 de (±8.5)	3.5 cd (±0.7)	7.9cde (±0.6)
**IR64**	9	3.7 f (±2.5)	0.3 e (±0.2)	11.0ab (±0.7)
**Rathu**	9	0.0 f (±0.0)	0.0 e (±0.0)	- a
**Babawee**	16	2.6 f (±1.3)	0.2 e (±0.1)	8.9bdc (±0.9)
**F1**	16	1.5 f (±0.65)	0.13 e (±0.05)	10.3abc (±0.8)
**Average**		42.2 ** (±10.8)	3.5 ** (±1.0)	7.7** (±0.8)

Means± SE within columns followed by the same letters are not significantly different (P*>*0.05, Duncan test).

** =  Significant at 1% probability level; *  =  Significant at 5% probability level; ns  =  Non-significant.

‘-‘  =  no honeydew observed in 12 h.

### Characterization of the EPG waveform feeding pattern for BPH on rice

**Figure 1:**
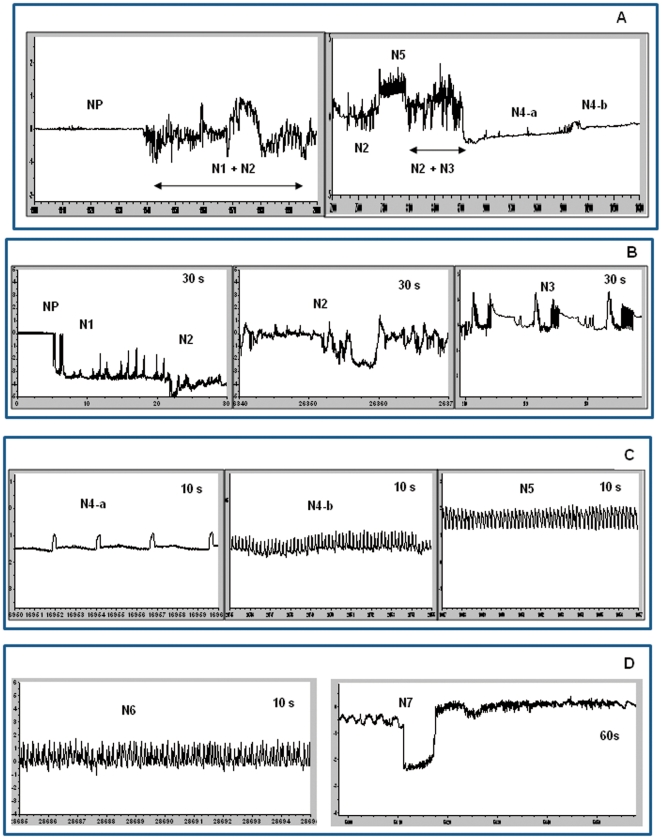
Classification of EPG waveform feeding pattern for BPH in rice. A: Overall typical waveform in two hours. B: Non penetration (NP), pathway (N1, N2 {irregular mixed} and N3 {transition phase before N4-b start} characterization in 30 seconds. C: Sieve element salivation (N4-a) , Phloem (N4-b) and xylem ingestion phase (N5) characterization in 10 seconds . D: Unclear waveform types; derailed stylet mechanics (N6) and potential drop (N7) characterization in 5 and 60 seconds.

Figure 1 shows a typical DC-EPG waveform pattern produced by BPH on rice based on the analyses of Kimmins [26], Lösel and Goodman [27] and Seo *et al* [21], and in this analysis non penetration (NP) waveform correlates with absence of feeding. In pathway phase the BPH stylets are inserted into the plant producing EPG waveforms that are irregular with increased amplitude. We identified three main EPG patterns (N1, N2 and N3) similar to those identified by Seo *et al* [21] (figure 1A). N1 waveforms were difficult to identify, appearing only for a few seconds. Generally, N2 waveforms appeared immediately after the NP waveform and consisted of waveform shapes of variable frequency and amplitude. N2 was usually followed by N3 in which the shape was consistent, but with a higher amplitude. Subsequently the N4-a waveform appeared. Unlike Seo *et al* [21], in the present study we combined the waveforms N1-N3 into one type, the pathway waveform . This helped us to reduce our experimental work load in the context of developing a relatively high throughput system. The N5 waveform occurred occasionally during the pathway period and the waveform had shown a consistent shape (figure 1C) close to that found by Seo *et al* [21]. Interestingly, this shape is also similar to aphid EPG xylem characterization [24]. Waveforms N6 and N7 also occur during pathway phases in our experiments, however these two types of waveform could not be correlated with those seen in other EPG studies. The N6 waveform pattern is similar to N5 but of higher frequency without the consistency of shape (figure 1D). We categorized this N6 waveform as ‘derailed stylet mechanics’ on the grounds that the pattern was similar to that noted by Tjallingii [30] for aphid feeding. Tjallingii [24], has also associated derailed stylet mechanics with a mechanical ‘error’ impeding the stylets forming a properly functioning bundle. Here we interpret our N6 waveform as representing penetration difficulties within the plant tissue generally [31]. The N7 waveform we classified as potential drops; the waveforms suddenly drop from active pathway activities (figure 1D). N7 waveforms are similar to those noted by Tjallingii [30] described for aphids where it is believed to correlate with cell penetration. N4-a and N4-b patterns are clearly (figure 1C) distinguishable from other waveforms and have been confidently attributed to the sieve element feeding phase [21], [26]. In addition, strong correlation between honeydew excretion and N4-b phase (Table 2) provides further evidence of phloem ingestion activity.

**Table 2:** Correlation coefficients and significance levels of qualitative EPG and honeydew drop characters among 12 rice accessions.

	Pathway	N4-a	N4-b	N5	N6	N7	Average Honeydew drops in 12 hours	Total Honeydew drops in 12 hours
**Non Penetration**	0.768**(0.003)	−0.700**(0.011)	−0.907**(<.001)	0.607**(0.036)	0.007 **^ns^**(0.984)	0.427 **^ns^**(0.166)	−0.735**(0.006)	−0.719** (0.008)
**Pathway**		−0.693**(0.012)	−0.947**(<.001)	0.450 **^ns^**(0.142)	0.107 **^ns^**(0.740)	0.662**(0.019)	−0.875**(<.001)	−0.857**(<.001)
**N4-a**			0.663**(0.019)	−0.474 **^ns^**(0.119)	−0.307 **^ns^**(0.332)	−0.654**(0.021)	0.835**(<.001)	0.833**(<.001)
**N4-b**				−0.625**(0.029)	−0.037 **^ns^** 0.903)	−0.629**(0.028)	0.837**(<.001)	0.815**(<.001)
**N5**					−0.149 **^ns^** (0.645)	0.726** (0.008)	−0.545* (0.067)	−0.519*(0.084)
**N6**						0.131 **^ns^**(0.684)	−0.096 **^ns^**(0.767)	−0.09 **^ns^**(0.781)
**N7**							−0.774**(0.003)	−0.771**(0.003)
**Average Honeydew drops**								0.996** (<.001)

** =  Significant at 1% probability level.

* =  Significant at 5% probability level; ns  =  Non-significant.

### Correlation of *N. lugens* feeding and honeydew production

**Figure 2:**
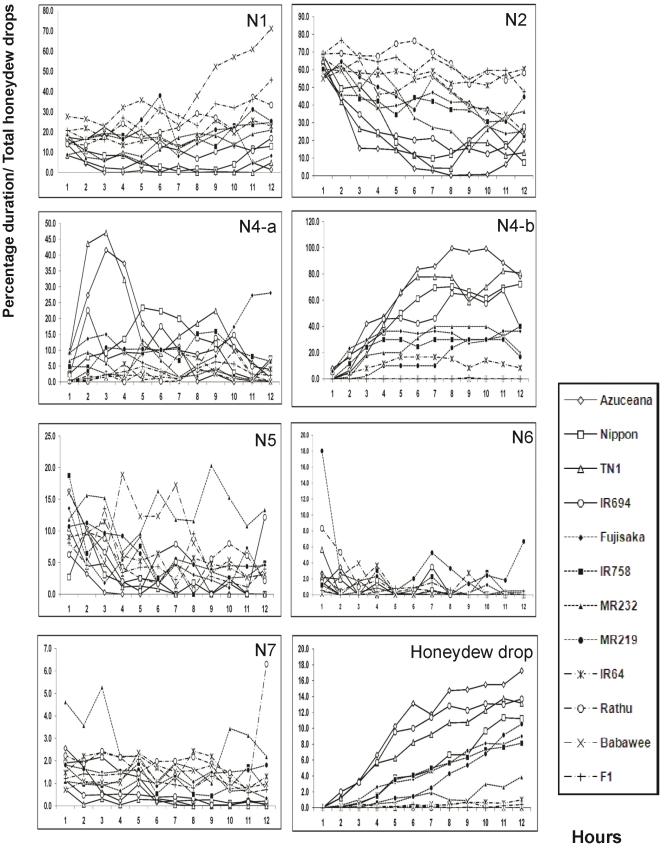
Comparative graph between EPG waveform and honeydew drops. This graphs are based on percentage duration for each waveform types, NP (Non penetration), pathway, N4-a (sieve element salvation), N4-b (phloem ingestion), N5 (xylem ingestion), N6 (derailed of stylet mechanics) and N7 (potential drop) and honeydew drops for 12 rice varieties. Data were recorded from the first time when BPH make a connection with the plant and then stopped after 12 hs.

Generally pathway activity (the sum of N1, N2 and N3 phases) decreased over the first 6 h of feeding with a concomitant increase in phloem sap ingestion (N4-b Figure 2). The increase in N4-b activity was paralleled by an increase in honeydew production. In some varieties (notably TN1 and Azucaena) there was an initial peak in N4-a activity (salivation) which declined during later stages of feeding. The other EPG waveforms did not show any clear pattern except for NP. Rice varieties Rathu, Babawee and F1 showed increase in NP percentage duration in the last three h of the 12 h feeding period.

Linear correlations between EPG waveforms and honeydew drop variables were calculated (table 2). Strong positive correlations were found between salivation (N4-a), phloem sap ingestion (N4-b) and honeydew drop production. Positive correlations were also found between non penetration, pathways (N1-N3) and cell penetration (N7) activities. In contrast, pathway behaviour showed a high negative correlation with N4-b waveform ( r = −0.947, P = <0.01), average rate of honeydew drop production (r = −0.875, P = <0.01) and total number of honeydew drops (r = −0.857, P = <0.001).

### Phloem location

The presence of the salivation waveform (N4-a) indicates the first time that the stylets encounter the sieve element. There was no significant difference in the time to the first N4-a waveform for BPH across all rice varieties (Table 3). BPH on Azucaena took the shortest time to reach the sieve element of 3.4 h and reached the phloem in a similar time when feeding on Nipponbare, IR694 and TN1. N4-b waveform represents phloem acceptance and successful phloem ingestion. There were significant differences in the time to the first N4-b waveform on the different rice varieties. Based on frequency of the N4-b waveform, BPH was unable to successfully ingest sieve element sap on Rathu Heenathi and Babawee. The qualitative differences between N4-a and N4-b timings indicate that BPH has a similar ability to locate the sieve element across all varieties but there is variation in the ability to successfully sustain phloem sap ingestion.

**Table 3:** Fastest time (h) to N4-a and N4-b waveform patterns within 12 h experiment.

Variety	n	N4-a	N4-b
**Azuceana**	7	1.0±0.2	3.4 d (±0.8)
**Nipponbare**	8	1.2±0.2	3.8 d (±0.7)
**TN1**	9	1.8±0.7	4.4 d (±0.8)
**IR694**	10	3.4±1.1	5.4 cd (±1.4)
**Fujisaka**	11	2.8±1.1	8.1 bc(±1.6)
**IR758**	10	3.4±1.1	8.3 bc (±1.6)
**MR232**	10	5.2±1.7	8.9 ab (±1.2)
**MR219**	10	5.6±1.2	10.1 ab (±1.0)
**IR64**	12	5.1±1.3	10.4 ab (±1.1)
**Rathu**	8	6.1±1.7	- a
**Babawee**	10	4.5±1.0	- a
**F1**	15	4.2±1.0	11.8 a (±0.2)
**Average**		3.7 ns±0.5	8.2** (±0.9)

Means± SE within columns followed by the same letters are not significantly different (P*>*0.05, Duncan test).

** =  Significant at 1% probability level; ns  =  Non-significant.

‘-‘  =  no N4-b waveform pattern observed in 12 h.

### Comparison of duration and frequency of EPG waveforms

The average percentage duration of seven EPG waveforms from BPH on the twelve rice varieties during the final 5 h of the 12 h feeding period was calculated (table 4). A Kruskal-Wallis nonparametric analysis indicated that all EPG activities varied significantly between rice varieties except for salivation (N4-a). BPH feeding patterns on Rathu Heenathi and Babawee were markedly different when compared to other varieties. For example on these two varieties BPH spent around 90% of time not penetrating (non penetration - NP) or in pathway. However no N4-b behaviour was observed. In contrast, BPH feeding on Azucaena showed the highest duration (92.5%) of phloem ingestion (N4-b) over this period. Table 5 shows the average frequency of all EPG waveforms in each h over the last 5 h of experiment. ANOVA revealed that phloem ingestion (N4-b) and derailed stylet mechanics (N6) were highest in TN1, IR694 and Nipponbare.

**Table 4:** Comparison of different EPG waveform feeding patterns of BPH on different rice varieties for 5 h (8–12 h) (percentage duration and standard error).

	N	NP	Pathway	N4-a	N4-b	N5	N6	N7
**Azuceana**	7	1.2e(±1.0)	5.7c (±4.1)	0.5 (±0.5)	92.5a (±5.1)	0.0c (±0.0)	0.0c (±0.0)	0.1ef (±0.1)
**Nipponbare**	8	6.4de (±5.5)	17.2bc (±8.0)	7.4 (±4.1)	67.9a (±14.2)	0.9bc (±0.6)	0.0c (±0.0)	0.2def (±0.1)
**TN1**	9	1.1e (±0.9)	13.2bc (±8.4)	11.9 (±5.5)	73.8a (±8.1)	0.0c (±0.0)	0.0c (±0.0)	0.0f (±0.0)
**IR694**	10	11.7cde (±8.3)	17.2bc (±6.5)	8.5 (±5.7)	58.6ab (±11.7)	3.8ab (±1.8)	0.0bc (±0.0)	0.2def (±0.1)
**Fujisaka**	11	13.7bcd (±4.5)	32.0abc (±8.4)	17.2 (±7.9)	32.8bcd(±14.2)	3.1abc (±1.2)	0.3bc (±0.2)	0.9abcd (±0.3)
**IR758**	10	20.3bcd (±10.5)	31.8abc (±11.0)	11.2 (±7.4)	32.5bc (±14.8)	2.8ab (±1.3)	0.7bc (±0.6)	0.8bcde (±0.4)
**MR232**	10	18.3cd (±9.9)	40.5ab (±11.5)	2.9 (±1.9)	34.5bc (±14.2)	1.5abc (±0.7)	0.4bc (±0.4)	1.9abcd (±0.8)
**MR219**	10	23.2abc (±7.2)	39.9ab (±9.0)	1.9 (±1.2)	26.1cde (±13.4)	4.4ab (±1.4)	3.1a (±1.0)	1.4ab (±0.4)
**IR64**	12	22.1abc (±9.1)	54.9a (±9.3)	5.3 (±1.8)	11.4 cde (±7.6)	4.1ab (±1.1)	0.6b (±0.3)	1.6a (±0.3)
**Rathu**	8	29.5ab (±10.6)	57.2a (±9.3)	6.3 (±5.3)	0.0e (±0.0)	4.5a (±1.3)	0.0c (±0.0)	2.5ab (±1.5)
**Babawee**	10	45.2a (±12.1)	45.5ab (±10.9)	2.1 (±0.9)	0.0e (±0.0)	5.6ab (±2.5)	0.1bc (±0.1)	1.5ab (±0.3)
**F1**	15	34.2ab (±8.6)	56.6a (±7.6)	4.2 (±1.7)	0.2de (±0.2)	3.9ab (±0.8)	0.1bc (±0.1)	0.9abc (±0.2)
**Average**		19.9** (±3.8)	34.3** (±5.2)	6.6ns (±1.4)	35.9** (±9.0)	2.9** (±0.5)	0.4** (±0.3)	1.0** (±0.2)
**Chi-square**		42.22	30.56	9.47	57.14	37.76	28.47	38.05
**Pr>Chi-square** **(Kruskal- Wallis P value)**		<.0001	0.0013	0.5787	<.0001	<.0001	0.0027	<.0001

Means± SE within columns followed by the same letters are not significantly different (P*>*0.05, Kruskal- Wallis and Duncan test).

** =  Significant at 1% probability level; ns  =  Non-significant.

**Table 5:** Comparison percentage of time for different EPG waveform feeding patterns of *N. lugens* on different rice varieties for 5 h (8-12 h). (Average percentage frequency and standard error).

	N	NP	Pathway	N4-a	N4-b	N5	N6	N7
**Azuceana**	7	5.4 cd (±3.63)	15.3 d (±7.23)	2.597 (±2.59)	72.1 a (±13.36)	0.0 b	0 0c	4.56 d (±2.96)
**Babawee**	10	30.3 a (±10.04)	38.7 abc (±4.99)	5.215 (±1.27)	0.0 f	3.13 a (±1.14)	0.12 c (±0.12)	22.49 ab (±4.14)
**F1**	15	28.0 a (±7.91)	42.0 ab (±4.53)	4.35 (±1.20)	0.05 ef (±0.05)	3.43 a (±0.75)	0.11 c (±0.11)	21.99 ab (±3.26)
**Fujisaka**	11	12.6 abcd(±3.76)	33.8 abc (±6.62)	4.85 (±1.65)	28.22 cde (±13.93)	3.78 a (±1.42)	0.60 bc (±0.52)	16.15 abc (±4.44)
**IR64**	12	16.5 abc (±7.66)	44.0 ab (±4.15)	3.71 (±1.54)	2.16 def (±1.51)	5.18 a (±1.94)	1.67 ab (±0.84)	26.83 a (±3.22)
**IR694**	10	16.3 abc (±5.13)	32.9 bdc (±5.76)	6.04 (±2.90)	32.20 bc (±12.08)	5.93 a (±2.47)	0.0 c	6.67 cd (±3.03)
**IR758**	10	19.9 abcd (±10.34)	24.1 bdc (±8.03)	8.28 (±6.55)	33.33 cd (±14.91)	2.21 ab (±1.00)	0.59 bc (±0.52)	11.57 bcd (±4.85)
**MR219**	10	14.6 ab (±2.77)	41.3 abc (±3.21)	3.55 (±1.79)	12.19 def (±7.79)	5.31 a (±1.57)	2.83 a (±0.89)	20.22 abc (±3.42)
**MR232**	10	16.3 abcd (±7.99)	38.2 abc (±9.15)	2.55 (±1.53)	22.73 cd (±13.02)	2.01 ab (±0.89)	0.42 bc (±0.34)	17.77 abc (±5.09)
**Nipponbare**	8	7.4 bcd (±3.43)	24.9 cd (±7.88)	7.41 (±2.84)	51.34 ab (±15.43)	1.75 ab (±0.88)	0.0 c	7.15 cd (±3.35)
**Rathu**	8	20.2 a (±4.51)	48.8 a (±3.06)	2.84 (±0.95)	0.0 f	4.56 a (±1.39)	0.0 c	23.62 ab (±4.62)
**TN1**	9	2.7 d (±2.10)	26.9 cd (±6.04)	11.77 (±4.24)	58.58 ab (±8.72)	0.0 b	0.0 c	0.0 d
**Average**		16.78** (±2.05)	35.18** (±1.85)	5.21 ns (±0.79)	23.20** (±3.37)	3.26** (±0.41)	0.56** (±0.14)	15.78**(±1.29)
**Chi-square**		28.01	28.74	9.20	60.42	25.70	37.52	38.83
**Pr>Chi-square** **(Kruskal- Wallis** **P value)**		0.0032	0.0025	0.6034	<.0001	0.0072	<.0001	<.0001

Means± SE within columns followed by the same letters are not significantly different (P*>*0.05, Kruskal- Wallis and Duncan test).

** =  Significant at 1% probability level; ns  =  Non-significant.

### Cluster analysis

**Figure 3:**
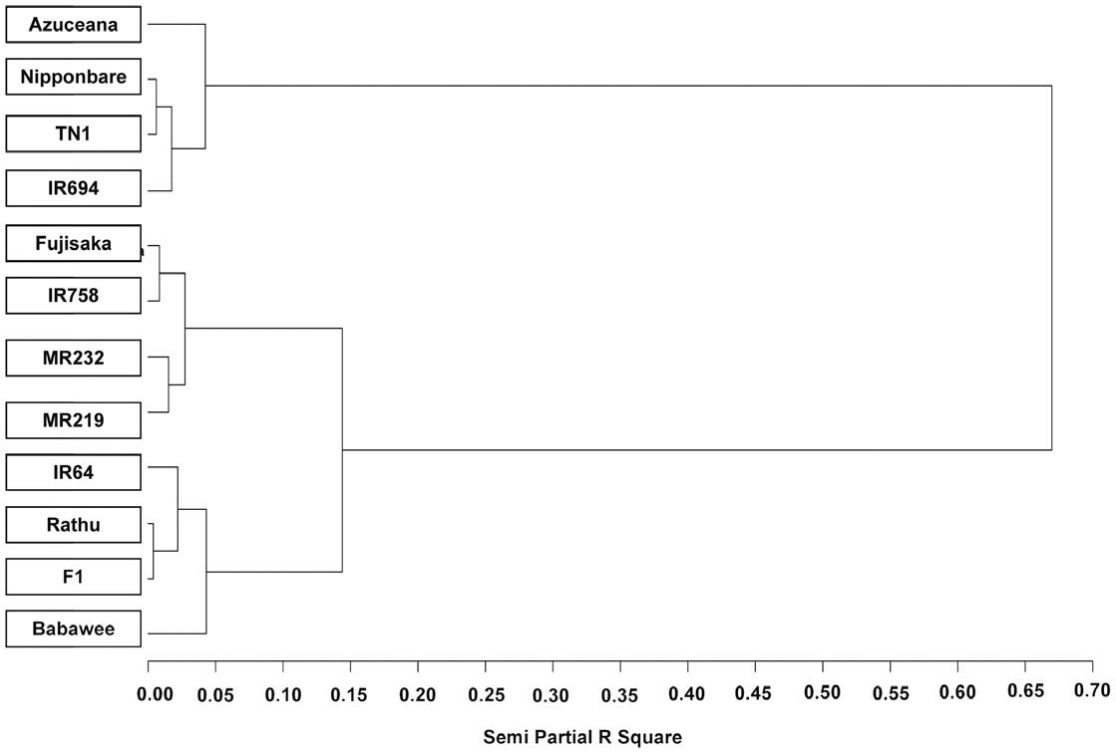
Dendrogram derived using Ward clustering on 56 characters (SAS, 2008). Twelve rice varieties have been divided into three different groups namely susceptible group 1(Azuceana, TN1, Nipponbare and IR694), moderately resistant group 2 (Fujisaka, IR758, MR232 and MR219) and strongly resistant group 3 (Rathu, IR64, Babawee and F1).

Cluster analysis using Ward's method based on Euclidean Distance was performed using 56 activities derived from EPG waveform duration and frequency for the last 5 h of the 12 h feeding period. Fundamentally, this multivariate method involves making pairwise comparisons of all objects (varieties), and then classifying them according to an average linkage method (Ward's) and illustrating the object relationships in a dendrogram [32]. Therefore, the real objective of this analysis is to summarize overall data for classification of resistant and susceptible varieties. Total and average honeydew data for the same last 5 h of feeding were also included in the analysis (table 6). The resulting dendrogram (figure 3) divided the 12 rice varieties into three main groups at a 0.15 semi partial R square value. Group 1 included Azucaena, TN1, Nipponbare and IR694. This group showed the greatest distance from the other two groups namely group 2 - Fujisaka, IR758, MR219 and MR232, while Rathu Heenathi, IR64, Babawee and F1 formed a third group.

**Table 6:** List of 56 characters used for cluster analysis and their significance levels from univariate test statistics using CANDISC procedure (SAS software).

No	Characters	Significance level (pr>F)
**1**	NP (average in 5 hours)	0.0016
**2**	Pathway (average in 5 hours)	<.0001
**3**	N4-a (average in 5 hours)	0.5787
**4**	N4-b (average in 5 hours)	<.0001
**5**	N5 (average in 5 hours)	0.0184
**6**	N6 (average in 5 hours)	0.1505
**7**	N7 (average in 5 hours)	0.0045
**8**	Average honeydew droplets in each hour	<.0001
**9**	Total honeydew droplets (average in 5 hours)	<.0001
**10**	Percentage frequency NP 1 (average in 5 hours)	0.0080
**11**	Percentage frequency Pathway (average in 5 hours)	0.0103
**12**	Percentage frequency N4-a (average in 5 hours)	0.3261
**13**	Percentage frequency N4-b (average in 5 hours)	0.0003
**14**	Percentage frequency N5 (average in 5 hours)	0.3225
**15**	Percentage frequency N6 (average in 5 hours)	0.2106
**16**	Percentage frequency N7 (average in 5 hours)	<.0001
**17**	NP (average in 8^th^ hour)	0.0006
**18**	NP (average in 9^th^ hour)	0.0010
**19**	NP (average in 10^th^ hour)	0.0179
**20**	NP (average in 11^th^ hour)	0.0073
**21**	NP (average in 12^th^ hour)	0.0162
**22**	Pathway (average in 8^th^ hour)	<.0001
**23**	Pathway (average in 9^th^ hour)	0.0017
**24**	Pathway (average in 10^th^ hour)	0.0017
**25**	Pathway (average in 11^th^ hour)	0.0002
**26**	Pathway (average in 12^th^ hour)	0.0201
**27**	N4-a (average in 8^th^ hour)	0.4602
**28**	N4-a (average in 9^th^ hour)	0.5107
**29**	N4-a (average in 10^th^ hour)	0.9851
**30**	N4-a (average in 11^th^ hour)	0.3991
**31**	N4-a (average in 12^th^ hour)	0.2513
**32**	N4-b (average in 8^th^ hour)	<.0001
**33**	N4-b (average in 9^th^ hour)	<.0001
**34**	N4-b (average in 10^th^ hour)	<.0001
**35**	N4-b (average in 11^th^ hour)	<.0001
**36**	N4-b (average in 12^th^ hour)	0.0002
**37**	N5 (average in 8^th^ hour)	0.3108
**38**	N5 (average in 9^th^ hour)	0.1659
**39**	N5 (average in 10^th^ hour)	0.2672
**40**	N5 (average in 11^th^ hour)	0.0139
**41**	N5 (average in 12^th^ hour)	0.5633
**42**	N6 (average in 8^th^ hour)	0.4917
**43**	N6 (average in 9^th^ hour)	0.4497
**44**	N6 (average in 10^th^ hour)	0.0187
**45**	N6 (average in 11^th^ hour)	0.1411
**46**	N6 (average in 12^th^ hour)	0.3181
**47**	N7 (average in 8^th^ hour)	0.0053
**48**	N7 (average in 9^th^ hour)	0.0004
**49**	N7 (average in 10^th^ hour)	0.0409
**50**	N7 (average in 11^th^ hour)	0.0106
**51**	N7 (average in 12^th^ hour)	0.2014
**52**	Honeydew drop (average in 8^th^ hour)	0.0003
**53**	Honeydew drop (average in 9^th^ hour)	0.0008
**54**	Honeydew drop (average in 10^th^ hour)	<.0001
**55**	Honeydew drop (average in 11^th^ hour)	<.0001
**56**	Honeydew drop (average in 12^th^ hour)	<.0001

**Figure 4:**
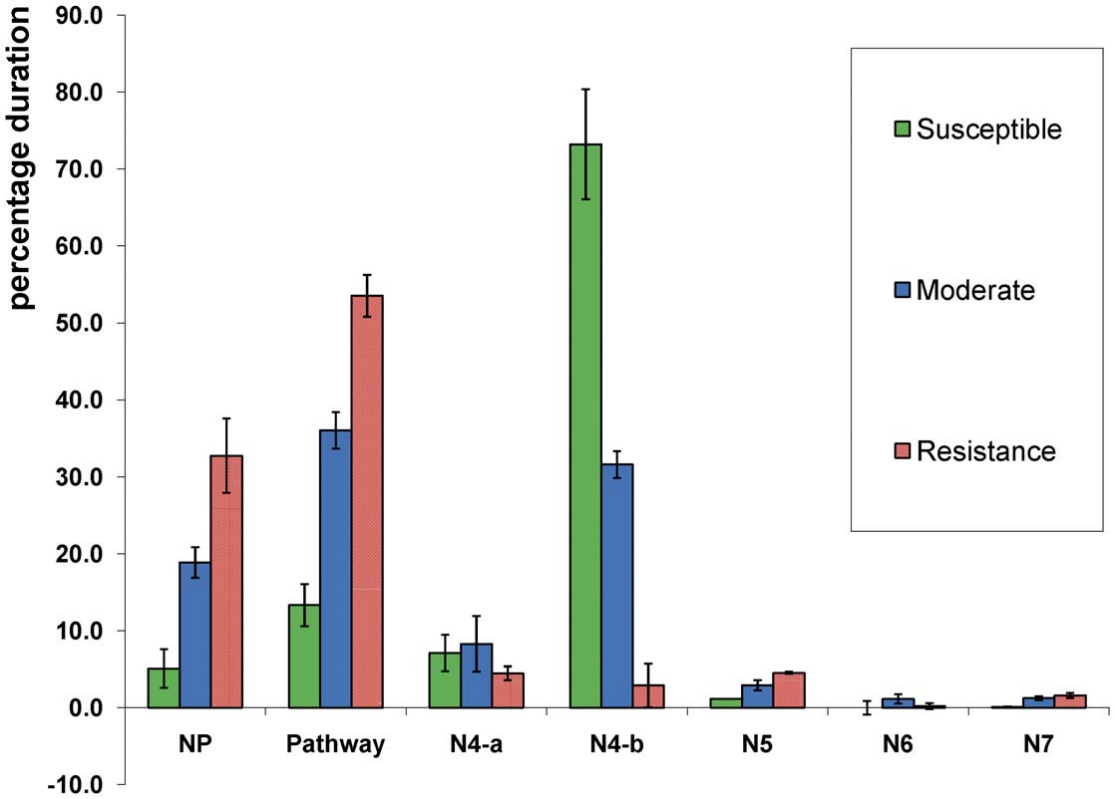
Average percentage duration of 7 types EPG waveform. The histogram graph are based on 8 to 12 h (5 hs) recording and followed the susceptible, moderate and resistance groups produced by cluster analysis.

In Univariate analysis (table 6), 38 out of 56 activities showed highly significant differences between varieties. These characters mostly related to non penetration, pathway, N4-b and honeydew drop, and the resistance versus susceptibility can clearly be distinguished for all 12 varieties. Further analysis of the common characteristics of the three groups identified by cluster analysis demonstrated that resistance was associated with high percentage duration of NP, pathway, N5, N6 and N7 EPG waveform characters (figure 4). In contrast the susceptible group was associated with the longest duration of N4-b (phloem ingestion). Interestingly N4-a (sieve element salivation) pattern waveform did not statistically differentiate between those groups.

## Discussion

In this study we have been able to characterize BPH feeding behaviour using DC- based electrical penetration graph (EPG), and use this to screen 12 rice varieties of differing resistance, facilitating the efficient and detailed classification of rice germplasm for insect resistance.

### Feeding patterns of BPH

We identified seven typical waveforms for BPH, more or less consistent with those previously described for BPH using DC-based EPG [21], [26], [27]. Seo *et al* [21] in their most recent histological study related to EPG and BPH stylet penetration have provided valuable and detailed information regarding waveform classification. Therefore, we chose their descriptions as our main guide for EPG characterization. Generally, the sequence of BPH feeding process will always start with non penetration (NP), and NP is the easiest waveform to describe. A straight line waveform indicates that no feeding activities are happening or that the stylet has still not inserted into the plant. Our second waveform however was complicated because it produced a variation of frequency, amplitude, voltage level and shape of waveform. Kimmins [26] classified this waveform into two phases, P2 and P3, while Seo *et al* [21] separated this into three types, N1 (penetration initiation), N2 (salivation and stylet movement) and N3 (extracellular activities). This irregular waveform pattern happens within epidermal and mesophyll cell membranes [27] in the pathway to the phloem. This is one reason we use to justify classifying these waveforms as one type, pathway. This has given us more confidence in our EPG classification, focuses only on our objective priority and is experimentally more time-efficient. The N4-a and N4-b are relatively simple to identify because their waveform patterns are consistent with those previously described by Seo *et al* [21]. N4-a always occurs just before N4-b appears. Seo *et al* [21] determined that at this stage, the BPH stylet tip was already located in the phloem region but no sap was actually ingested. Seo *et al* [21] claim N4-a is related to intracellular activity in the phloem region on the basis of the different signal amplitude and frequency (Figure 1A) compared to pathway. This phase is close in character to the E1 waveform type (sieve element salivation phase) in aphid studies [33] which also was described on the basis of stylet position, level of voltage, waveform shape and absence of honeydew drops [21]. By contrast, the duration of N4-b shows a critical difference to the N4-a waveform, being generally sustained over long periods. Associated with N4-b, honeydew drops were produced, providing strong evidence that BPH were ingesting phloem sap at this time.

The other three waveforms, N5, N6 and N7 appear irregularly from time to time during pathway period. N5 waveform is similar to P5 described by Kimmins [26] and type II waveform by Lösel and Goodman [27]. These authors suggest that this waveform is associated with xylem ingestion [21]. We have noted a waveform, N6, not described by other authors; this waveform pattern appears similar to N5 but with much higher repetition and frequency and inconsistent shape. Accordingly, we have classified N6 as ‘derailed stylet mechanics’ on the basis of its similarity with the waveform described for aphids [24] and we associate it with penetration difficulty. Kimmins [26] suggests that the BPH stylet does not puncture cell membranes during the pathway phase leading to the absence of the characteristic cell penetrations of pathway phase in aphid studies. However, in the present study apparent cell penetrations (N7) could be clearly identified (Figure 1D). The discrepancy between the two studies may be attributed to the low input impedence of the EPG amplifier in the previous study [27].

BPH feeding can be divided into two main categories based on the EPG waveforms [23]. The first represents to non-ingestion activities, beginning when the BPH first touches the plant, followed by the movement of stylet tip into the plant through the cell wall, epidermal and mesophyll cell membranes until the stylet reaches the phloem region. EPG waveforms NP, Pathway, N5, N6 and N7 are included in the first category. In the second category, we include EPG N4-a and N4-b waveforms as ingestion activities. Correlation analysis based on the full 12 h feeding period presented in table 2 indicates a strong relation between these two categories. There was a high positive correlation between N4-a, N4-b and honeydew production but a high negative correlation with NP, pathway, N5, N6 and N7 EPG waveforms. Therefore, a higher proportion of time in the first waveform category is consistent with higher plant resistance to BPH while more time spent in category two is associated with susceptibility.

In most rice varieties the total duration of pathway phase decreased after 3 to 4 h and then remained constant over the remaining 8 h. The average time in all 12 rice varieties for BPH to reach N4-b waveform, and then to start to produce honeydew were 8.2 and 7.7 h respectively.

To focus on varietal differences in category 2 activities, comparisons between rice varieties were performed on the last 5 h of feeding (8–12 h). Using this subset of data, evaluation of resistance in the twelve rice varieties was different to previous reports where data were included from h zero as in Seo *et al* [21], Hattori [23] and Kimmins [26].

### Differentiation of resistance and susceptibility

BPH clearly responds differently to different rice varieties spending more than 80% of its time exhibiting the non-ingestion waveform types such as non-penetration or pathway in the varieties previously identified as resistant by Brar *et al* [34] (Rathu Heenathi , Babawee and IR64). A similar result of resistance characterization based on EPG was also found by previous researchers using other varieties such as IR56 [3], ASD7 [35] and IR 62 [26]. However, in susceptible varieties such as TN1 (commonly used as a control variety in many BPH experiments), BPH ingested phloem sap for a long period without interruption. Therefore a longer duration for N4-b waveform could easily be found. Interestingly, N4-a salivation activity for last 5 h period was not significantly different between resistant and susceptible rice varieties, indicating that BPH could reach the sieve element region in both resistant and susceptible, but could only ingest the phloem sap in susceptible genotypes. These results support the suggestion of Hattori [23] that resistance to BPH is determined by phloem related characters. Phloem based resistance may have its basis in phloem chemistry [20], [36] where silicic, oxalic [36], [37], [38], [39] and phenolic acids [36], [40], sterols [41] and apigenin-C-glycosides [42], [43] have been implicated in resistance to BPH. The low level of essential amino acids in the phloem could influence BPH feeding [20] perhaps representing phago-stimulatory cues. The interaction of plants and herbivorous insects is complex [44] and still not well understood and further advances may require molecular approaches [44].

A clear picture of resistance based on EPG waveform and honeydew drop data has been presented by cluster analysis. The twelve rice varieties could be classified into three groups. Group 1 was classified as the susceptible group because the average percentage duration of N4-b EPG waveform (category 2) was found to be the highest. In contrast, EPG waveform NP, pathway, N5 and N6 of group 1 showed the lowest values. These results clearly indicate that BPH could easily feed on the phloem sap in this group. As we expected, the common control rice variety, TN1 was classified in this group 1. The other three varieties in the susceptible group are Azucaena, Nipponbare and IR694. Groups 2 and 3 have a much closer relationship, but with group 3 being more resistant than group 2. Consistent with this, the varieties in this group have previously been found to contain the resistance genes *bph1* in IR64 [45], *bph4* in Babawee and *bph3* in Rathu Heenathi [46], [47] and the F1 (from the cross between Rathu Heenathi and TN1). BPH spent more time in the non-feeding phase whether in NP, pathway or occasionally in N5 waveform (xylem), possibly to overcome dehydration [48]. This result was found to be slightly different to that of Cohen *et al* [45]. Although IR64 was classified as resistant, their values for N1+N2+N5 and N6 are the lowest in that group. In addition, our experiment was conducted under full environment control (temperature and relative humidity) which highly influences BPH behavior [49]. Furthermore, the Cohen *et al* [45] classification covered a greater number of parameters including fecundity, nymph survival, feeding rate and an antixenosis test. Our parameters are more specific to BPH feeding ability with the limitation of the 12 h period.

The moderately resistant group 2 contained another four varieties namely MR232, MR219, Fujisaka and IR758. There is very limited information available on their genetic backgrounds but they are all products of a long history of breeding, with ancestors a likely source of some resistance genes contributing to their moderate resistance, and at least one of the parents of MR219 and MR232 is known to have possessed insect resistance [Habibudin 2009, pers. comm., 21 Nov, [50].

This study has provided new information on the mechanism of resistance to BPH on 12 rice varieties. The results confirmed and extended previous research using the EPG method to quantify BPH feeding behaviour on rice, and allowed the twelve rice varieties to be unequivocally divided into three groups; susceptible, moderately resistant and highly resistant. This study has demonstrated that BPH has the ability to locate the sieve elements of the different varieties, but there is variation in the ability to begin phloem sap ingestion thus providing a potential explanation for resistance in these varieties. Future work should focus on the underlying mechanisms at the molecular level. The relatively high-throughput, rapid and inexpensive method of screening germplasm used here can be utilized to identify in genetic resources collections natural sources of genetic variation conferring resistance to BPH in rice, and almost certainly for other pest/crop combinations as well. A firm platform for further genomic and transcriptomic studies to reveal candidate genes for the resistance has also now been established.

## Materials and Methods

### Plant material

The rice varieties used in this study and their origin are presented in table 7. Seeds were provided by IRRI (International Rice Research Institute) and MARDI (Malaysia Agriculture Research and Development Institute). The F1 is derived from a cross between Rathu Heenathi and TN1 developed in 2008 by MARDI. All seeds were germinated in Petri dishes on filter paper and then transferred to 5 cm diameter pots containing multipurpose compost (HUMAX). Plants were then maintained in a plant growth room at 24±3 C0 with 60±10% humidity and L16:D8 photoperiod. Plants aged between 40–50 days [51] were used for experimentation.

**Table 7:** List of rice varieties and their origin used in this study.

No	Variety	Accession numbers	Origin	Resistancelevel	Reaction to biotype1 2 3 4	References
1	TN1	11000 (MARDI)	Taiwan	Susceptible	S S S S	4
2	Azucaena	351438 (IRRI)	Japan	Susceptible	S S S S	44
3	Nipponbare	318852 (IRRI)	Japan	Susceptible	S S S -	50
4	IR694	777182 (IRRI)	Philippines	Unknown	- - - -	-
5	MR232	12047 (MARDI)	Malaysia	Moderate	- M - -	48
6	MR219	11633 (MARDI)	Malaysia	Moderate	- M - -	48
7	IR758	1876352 (IRRI)	Philippines	Unknown	- - - -	-
8	Fujisaka	00444 (MARDI)	Japan	Unknown	- - - -	-
9	IR64	50533 (IRRI)	Philippines	Resistance	S M S S	44
10	Rathu Heenathi	07637 (MARDI)	Sri Lanka	Resistance	R R R R	4, 48
11	Babawee	06246 (MARDI)	Sri Lanka	Resistance	R R R R	4, 48, 49
12	F1 (Rathu X TN1)	New (MARDI)	Malaysia	Unknown	- - - -	-

### Insect culture

Brown planthopper (BPH) biotype 2 cultures were obtained in July 2008 from MARDI research station at Pulau Pinang, Malaysia. These BPHs were then transferred to a mature TN1 rice clone and kept in net cages in an insect growth facility with similar conditions to above. The host plants were changed every month. Only brachypterous adult females were selected for experiments.

### Honeydew clock

The rate of honeydew drop production was measured using the modified methods of Wilkinson and Douglas [52] and Daniels *et al* [48]. Honeydew drops were collected from individual BPH on filter paper treated with 0.1% bromophenol blue (Sigma-Aldrich Company Ltd., UK) and 0.01 M HCl Sigma-Aldrich Company Ltd). This treatment generates a yellow paper that turns blue when in contact with aqueous solutions such as honeydew droplets. Treated filter paper was placed on a plastic Petri dish circle plate attached to the h spigot of a clock such that it rotated 3600 over 12 h duration. A rice plant was clamped horizontally over the disk. BPHs were starved for one h before use and then introduced to the plant, positioned so that the honeydew produced dropped directly onto the treated filter paper. The frequency of honeydew drop production was calculated after a 12 h period. Data were collected for analysis when BPH produced honey dew for more than 3 h after the start for the experiment.

### EPG Technique

BPH feeding behaviour was recorded and classified using a GIGA-8 DC electrical penetration graph (EPG) amplifier system introduced by Tjallingii [24], [30]. Only adult brachypterous females [23], [27], [,51] were selected from the insect cage based on their size and active behaviour. BPHs were cooled to −20oC for 60 s and then carefully connected to a 3 cm length of 18.5 µm diameter gold wire (EPG system, Wageningen University) with conductive silver glue on their dorsum. After a 1 h starvation period, the BPH were then linked to a GIGA 8 model DC-EPG amplifier (EPG system, Wageningen University). To complete the electronic circuit, they were then connected to a stem area of each rice plant between1−2 cm above the soil at internode 2 or 3. The experiment was conducted in the insect culture room at 24±3 C0 with 60±10% humidity under a continuous photoperiod. In order to reduce technical error, recordings were only made on 4 channels simultaneously. Probing behaviour was recorded for 12 hs continuously. At least 7 replicates per rice variety were obtained. All recorded signals were analysed using probe 3.4 software versions (Wageningen Agricultural University, 2007).

### Statistical analyses

EPG waveform characterization namely NP (non penetration), pathway, N4-a (sieve element salivation), N4-b (phloem ingestion), N5 (xylem ingestion), N6 (derailed stylet mechanics) and N7 (cell penetration) were identified as decribed by Tjallingii [30], Vellusamy and Heinrich [3], Seo *et al* [21,], Kimmins [26], Lösel and Goodman [27], Hattori [23], and Hoa *et al* [28]. Each feeding behaviour was expressed as a percentage of the total h and their frequency either for the whole 12 h experimental period or the final 5 h period (8–12 h). All summarising statistics were produced using Excel. SAS version 9.1 (SAS Institute, 2008) was used for more detailed statistical analysis such as PROC ANOVA for the analysis of variance (ANOVA) and comparison of treatment means (Duncan). However this analysis was only used for the parameters of honeydew drops and fastest time N4a and fastest time N4b EPG types within the 12 h experiment. The PROC NPAR1WAY procedure for the Kruskal-Wallis test was used for the parameters of percentage duration and frequency of each waveform type. This nonparametric statistical analysis is often used for a suspected non-normal population [53]. Mean comparisons of each parameter were conducted using Duncan's multiple range test (P<0.05). For correlation analysis, PROC CORR was conducted on the 12 h experiment to identify the relationships between parameters in this study. Finally, PROC CLUSTER and PROC TREE were used to evaluate the relationship between all 12 varieties. The Euclidean distance coefficient and Ward's method (1963) [54] were selected for the cluster analysis.
